# Meet the editors: Nico Vervoort

**DOI:** 10.1002/ansa.202100003

**Published:** 2021-01-23

**Authors:** Nico Vervoort, Paul Trevorrow

**Affiliations:** ^1^ Janssen Pharmaceutica NV Turnhoutseweg 30 Beerse B‐2340 Belgium; ^2^ Wiley, The Atrium Southern Gate Chichester West Sussex PO19 8SQ UK

Nico Vervoort received his PhD degree in Applied Bioengineering in 2004 from the Vrije Universiteit Brussel under the guidance of Prof. Gert Desmet where he studied the use of shear‐driven chromatography and application of computational modeling in the design of novel microfluidic column formats and stationary phases. In 2005, he joined Janssen Research and Development as a Scientist in the Chemical Process Control group, the analytical group within the small molecule chemical development organization. In 2013, he joined the preparative chromatography group at Janssen as a Senior Scientist, becoming group leader in 2018. He is now Science Lead within the Janssen Process Analytical Research group. He is an author of 24 scientific publications and one patent application in the field of analytical chemistry.


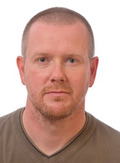


**An interview with Nico**

**Would you briefly explain your responsibilities?**

I am co‐responsible for setting the analytical strategy for projects in the small molecule API development area. The group is called the "Process Analytical Research" group and besides project work my role is to support introduction of novel analytical techniques and manage external collaborations related to analytical technology.

**Why did you choose a career in analytical chemistry?**

I have always had an affinity with science, especially with chemistry and biology, which eventually led me to study bioengineering. Over the different courses and lab work you come into contact with a diverse set of analytical tools that help you find answers so the questions you are trying to solve so that's when I picked up an interest that stuck.

**Of all your research projects, which one was your favourite and why?**

That would be the co‐development of a concept called "Shear‐Driven Chromatography" in the group of Prof. Desmet during my PhD. At that time the group was just getting started and we had to start from scratch with limited funds so we needed to be very creative in finding solutions using simple and available technology, which also meant we had a lot of freedom. In addition, the people in the group just were a good match and we had a lot of fun during that time.

**What is your vision as editor on *Analytical Science Advances*?**

Important for me is that we strive to create a journal of high quality that helps to lower the barrier in the dissemination of knowledge and sharing of data. There should be room for interdisciplinary topics on the state‐of‐the‐art in analytical science by which we can also differentiate the journal from others.

**What do you think is the key to success in a scientific career?**

Being curious, having a problem‐solving attitude combined with the necessary perseverance.

**Who were the most influential people in your career?**

Without a doubt, and this probably goes for a few people, that would be Gert Desmet under whose guidance I obtained my PhD.

**As a mentor and advisor, what do you advise your students in general?**

Being rigorous and apply critical thinking, be open to different perspectives and try to look at a problem from different angles. Take ownership of what you are doing and don't always rely on others to provide a solution or an answer to a problem.

**What do you consider to be the more exciting topics in analytical chemistry?**

Being in industry I'm always curious on any evolution of analytical tools, allowing us to generate better or more data in shorter timeframes. Anything related to automation and data management or high‐throughput catches my attention.

**What are your views on the future of your field?**

I see the field is becoming more interdisciplinary, for example if we look at the advances being made in in‐silico modeling, simulation of chromatographic processes, and handling of large datasets.

**What are your favourite past‐times outside of science?**

Besides spending time with my family, reading, and listening to music, I'm an avid cook and like to go out and enjoy a good meal and a fine wine, but that's been a while given the current restrictions. During summertime, when weather permits, I like to go fishing as well.

**What would you do if you had 1‐year paid leave?**

Hmmm, a very theoretical situation I'm afraid but probably travel the world with my family.

**What nonscientist inspires you the most?**

These days I think we can only admire and feel inspired by all the doctors and nurses dealing with the pandemic and taking care of patients.

**What is in your pockets right now?**

Nothing now but most of the time it would be my mobile phone, car keys, and wallet.

**What is the first thing you do when you wake up?**

Since I'm a bit of a coffee addict the first thing I do after waking up is to have a cup of coffee.

**Have you enjoyed this interview?**

Very much, thanks.

